# Fabrication of Black Silicon Microneedle Arrays for High Drug Loading

**DOI:** 10.3390/jfb14050245

**Published:** 2023-04-26

**Authors:** Wei Cheng, Xue Wang, Shuai Zou, Mengfei Ni, Zheng Lu, Longfei Dai, Jiandong Su, Kai Yang, Xiaodong Su

**Affiliations:** 1 Jiangsu Key Laboratory of Thin Films, School of Physical Science and Technology, Soochow University, Suzhou 215006, China; 20204208004@stu.suda.edu.cn (W.C.); mfni@stu.suda.edu.cn (M.N.); luzh@suda.edu.cn (Z.L.); 20227908001@stu.suda.edu.cn (L.D.); xdsu@suda.edu.cn (X.S.); 2 Department of Burn and Plastic Surgery, Suzhou Hospital Affiliated to Nanjing Medical University, Suzhou 215000, China; 2022121959@stu.njmu.edu.cn (X.W.); jiandongsu@njmu.edu.cn (J.S.); 3 Suzhou Xiangbang Biotechnology Co., Ltd., Suzhou 215006, China

**Keywords:** transdermal drug delivery, silicon microneedle, black silicon, Ag-catalyzed chemical etching, drug loading capacity

## Abstract

Silicon microneedle (Si-MN) systems are a promising strategy for transdermal drug delivery due to their minimal invasiveness and ease of processing and application. Traditional Si-MN arrays are usually fabricated by using micro-electro-mechanical system (MEMS) processes, which are expensive and not suitable for large-scale manufacturing and applications. In addition, Si-MNs have a smooth surface, making it difficult for them to achieve high-dose drug delivery. Herein, we demonstrate a solid strategy to prepare a novel black silicon microneedle (BSi-MN) patch with ultra-hydrophilic surfaces for high drug loading. The proposed strategy consists of a simple fabrication of plain Si-MNs and a subsequent fabrication of black silicon nanowires. First, plain Si-MNs were prepared via a simple method consisting of laser patterning and alkaline etching. The nanowire structures were then prepared on the surfaces of the plain Si-MNs to form the BSi-MNs through Ag-catalyzed chemical etching. The effects of preparation parameters, including Ag^+^ and HF concentrations during Ag nanoparticle deposition and [HF/(HF + H_2_O_2_)] ratio during Ag-catalyzed chemical etching, on the morphology and properties of the BSi-MNs were investigated in detail. The results show that the final prepared BSi-MN patches exhibit an excellent drug loading capability, more than twice that of plain Si-MN patches with the same area, while maintaining comparable mechanical properties for practical skin piercing applications. Moreover, the BSi-MNs exhibit a certain antimicrobial activity that is expected to prevent bacterial growth and disinfect the affected area when applied to the skin.

## 1. Introduction

Transdermal drug delivery (TDD) is an attractive strategy for delivering therapeutic agents due to its ability to avoid the challenges associated with traditional oral administration and hypodermic injections, such as inducing gastric irritation and liver injury, low bioavailability, and poor patient compliance [1,2]. One of the challenges in the context of TDD lies in overcoming the stratum corneum (SC), the outermost protective barrier of the skin. This barrier poses a significant hindrance to the effective delivery of active ingredients and limits the range of drugs that can be efficiently administered through TDD [3]. Various methods have been developed to enhance permeability through the stratum corneum barrier to improve TDD, including the use of chemical enhancers, iontophoresis, microdermabrasion, laser ablation, and electroporation. However, these techniques entail the risk of skin damage and require expensive equipment, which limits their practical application [4,5,6,7,8]. The aforementioned constraints can be surmounted by microneedles (MNs), a technology that can facilitate the delivery of various therapeutic compounds in a less intrusive manner with minimal damage to the skin while avoiding any potential harm to neurons within the dermis [9]. Compared to existing transdermal delivery strategies, MN-mediated TDD also facilitates flexible and painless topical administration, and can even achieve self-administration [10,11,12].

Coated MN systems for TDD have garnered considerable interest in both academia and the pharmaceutical industry due to their minimal invasiveness and ease of processing and application. A broad range of materials, including small molecules, biotherapeutics, and drug particles, can be applied to the surface of solid MNs as solid films, enabling their administration via this route [13,14]. After insertion into the skin, the drug agent dissolves and diffuses into the systemic circulation over time, and the MN system is subsequently removed from the insertion site. Moreover, the miniaturized size of the needles reduces the risk of medical complications during the application, such as nerve irritation, acute pain, or skin damage [15,16]. Transdermal administration via coated MNs offers significant benefits in terms of drug action and patient comfort compared to oral and parenteral administration [17]. However, at this stage, precise dose control, content uniformity, and the ability to mass-produce coated MNs remain bottlenecks that need to be addressed before commercialization [18,19,20].

For these reasons, research in this field is currently focused on the development and optimization of coating processes that can produce a reproducible and homogeneous coating on each MN arranged on the patch. Spray-, drip-, and dip-coating processes are capable of coating MNs in a high-volume production manner, but they can lead to significant drug wastage since the coating is deposited over the entire MN patch [21]. Custom masks with holes have been used to protect the substrate from the coating, but this also results in the deposition of the drug on the mask, which further limits the ease of delivery [22]. In recent years, dip-coating and inkjet printing have been developed to selectively generate precise drug coatings on MN shafts that are inserted into the skin, thus ensuring that a high proportion of the coated therapeutic dose is delivered [23,24]. Nonetheless, the primary constraint of dip-coating methodologies is their typical reliance on the inclusion of tackifiers, surfactants, or other excipients in the coating precursor solution to establish a dependable coating on the MN shafts. Such agents not only curtail the drug delivery capacity of the MN patch but also hold the potential to jeopardize its safety profile [25]. Although inkjet printing enables the active substance to be injected directly onto the needle surface in the form of tiny droplets, multiple coating cycles are required to cover the desired area of the MNs. This complex and time-consuming manufacturing process limits the ease of delivery and increases the economic burden [26].

MNs can be made with different types of materials, such as silicon [27,28,29], metal [30,31,32], biodegradable polymers [33], and glass [34]. Among these materials, silicon is widely utilized due to its availability, low cost, biocompatibility, and ease of fabricating simple [35,36,37,38] or complex structures [39]. However, current silicon MNs are usually fabricated using the process of a micro-electro-mechanical system (MEMS), including photolithography [40], deep reactive ion etching [41], and wet chemical etching (in combination with UV lithography and deep reactive ion etching) [42], which is expensive and not suitable for large-scale manufacturing and application. In addition, a Si-MN has a smooth surface, which makes it difficult to achieve high-dose drug delivery.

Herein, we report a solid strategy to fabricate a novel black silicon MN (BSi-MN) patch with ultra-hydrophilic surfaces for high drug loading. The BSi-MN patch fabrication involved two steps. First, plain Si-MNs were prepared via a simple method consisting of laser patterning and alkaline etching, which is much simpler than the conventional MEMS method. Second, nanowire structures were then prepared on the surfaces of the plain Si-MNs to form BSi-MNs through Ag-catalyzed chemical etching. The effects of preparation parameters, including Ag^+^ and HF concentrations during Ag nanoparticle deposition and [HF/(HF + H_2_O_2_)] ratio during Ag-catalyzed chemical etching, on the morphology and properties of BSi-MNs were investigated in detail. Furthermore, the BSi-MN patch was preliminarily tested for drug loading and antibacterial activity.

## 2. Materials and Methods

### 2.1. Materials and Chemicals

In this study, a <100>-oriented Cz-grown 8-inch single-side polished B-doped wafer with a starting thickness of 725 μm (Kunshan Xiaofei Photovoltaic Technology Co., Kunshan, China) was used as the substrate. The chemical reagents used in this study were potassium hydroxide (KOH, Aladdin, Shanghai, China, 99.9%), hydrofluoric acid (HF, Aladdin, Shanghai, China, 49%), silver nitrate (AgNO_3_, Macklin, Shanghai, China, 99%), hydrogen peroxide (H_2_O_2_, Macklin, Shanghai, China, 30%), nitric acid (HNO_3_, CrystalClear, Suzhou, China, 69%), propidium iodide (PI, Absin, Shanghai, China, 50 μg/mL), and 4′,6-diamidino-2-phenylindole (DAPI, Biogo, Shanghai, China, 10 μg/mL). None of the above reagents required further purification. Deionized water (DIW resistivity of ~18 MΩ·cm) and phosphate-buffered solution were used throughout the whole experimental process.

### 2.2. Preparation of BSi-MN Arrays

**Figure 1:**
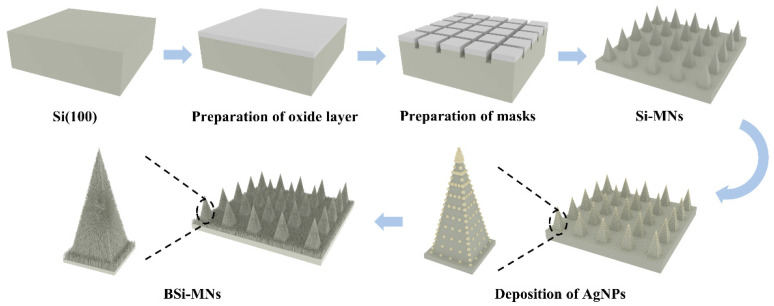
Schematic diagram of the formation of the BSi-MN array.

Figure 1 shows a schematic diagram of the process flows to fabricate the BSi-MN arrays. The <100>-oriented silicon wafer was first oxidized in an oxidation furnace tube through a wet oxidation process to prepare a protective layer of silicon oxide (SiO*_x_*) with a thickness of 1~1.5 µm on both sides. The protective layer on one side was opened by using a fiber laser (RPP12, DelphLaser, Suzhou, China) to form an orthogonal grid pattern. The detailed laser parameters were as follows: a speed of 100 mm/s, a power output of 25 W, a frequency of 5 KHz, and a duty cycle of 35%. Subsequently, alkaline etching was carried out in a 20 wt% KOH solution at 8 °C to prepare plain silicon MNs. The 2 × 2 cm^2^ Si-MN patches were obtained from the 8-inch Si-MN wafer through laser cutting. The Si-MN patches were cleaned using a 5 wt% HF solution to remove the surface oxide layer. Then, these Si-MN patches were immersed in a AgNO_3_/HF aqueous solution at room temperature for 20 s to deposit Ag-NPs on their surfaces. The subsequent formation of BSi-MNs was performed in an HF/H_2_O_2_ aqueous solution through a Ag-catalyzed chemical etching process. Finally, the BSi-MN patches were sequentially cleaned in 30 wt% HNO_3_ and dilute HF aqueous solutions to remove Ag residues and the oxide layer, and then dried. Throughout the wet process, the patches were thoroughly rinsed with DIW between all steps.

### 2.3. Quantification of Drug Loading

To determine the actual drug loading capacity of the BSi-MN patches, weighing experiments were conducted on the Si-MN patches and BSi-MN patches with an area of 2 × 2 cm^2^. Specifically, normal saline was first added to the surfaces of both patches using a syringe until excess, and the patches were then gently vertically lifted to remove excess normal saline. The weight of the MN sample is subtracted from the weight obtained by weighing to obtain its actual loading capacity.

### 2.4. Live/Dead Staining Experiments

In order to determine the antimicrobial capacity of the patches, live–dead bacterial staining using the fluorescent dyes PI and DAPI was performed in this study [43,44]. The standard strain *Escherichia coli* (*E. coli*) ATCC 25922 (ATCC, Manassas, VA, USA) was used in this experiment. The strain was incubated in a bacterial incubator at 37 °C until it reached the midlogarithmic phase. The optical density of the bacterial solution was adjusted to OD_600_ nm = 0.1. The Si-MN patches, including the plain Si-MN patches and BSi-MN patches, were immersed in 1 mL of the bacterial solution and incubated for 30 min in the dark. After incubation, all the patches were rinsed three times with phosphate-buffered solution and then stained successively with PI and DAPI. The patches were observed under an inverted confocal microscope (LSM 710, Zeiss, Jena, Germany) with a 63× oil objective. All images were taken under the same instrument settings. The fluorescence images were analyzed using ImageJ (version 1.51j8) software. We performed at least three independent experiments in each case.

### 2.5. Characterization

The morphologies of the patches were observed using a field emission scanning electron microscope (FE-SEM, Hitachi, S-4700). A contact angle measuring instrument (Shanghai Huafu Information Technology Co., Ltd., Shanghai, China, A23-605L) was employed to determine the contact angle. The reflectance spectra in the wavelength range of 400–1000 nm were measured using an ultraviolet–visible near-infrared spectrophotometer (Shimadzu, Kyoto, Japan, UV-3600). A precision mechanical tester (Tengba, Shanghai, China, Universal TA) was used to determine the mechanical properties of the MNs by using a probe to vertically compress the MN patch until the needle broke/collapsed. Fluorescence staining tests for live/dead bacteria were performed on Si-MNs and BSi-MNs using a laser scanning confocal microscope (LSCM, Zeiss, LSM 710). The size distributions of Ag-NPs deposited on the surfaces of Si-MNs were analyzed using NanoMeasure1.2. The coverage of Ag-NPs and the porosity of black silicon nanowire structures were calculated using ImageJ (version 1.51j8) software.

## 3. Results and Discussion

### 3.1. Fabrication of Si-MNs

**Figure 2:**
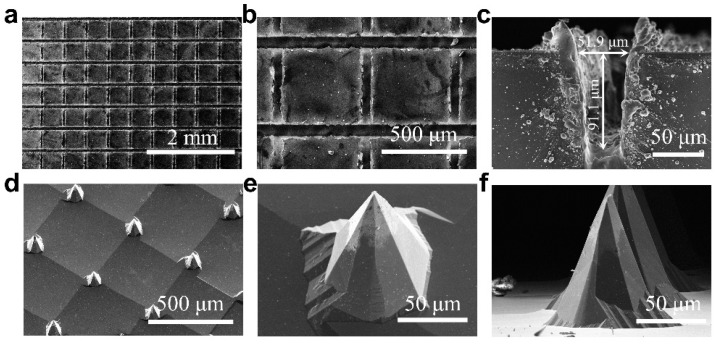
(**a**) Top-view SEM image and (**b**) enlarged-view image of the surface morphology of the wafer with the SiO*_x_* protective layer after laser grooving. (**c**) Cross-sectional SEM image of the laser kerf. (**d**) SEM image of the as-prepared Si-MN array at 30° tilt. (**e**) SEM image of a single Si-MN at 30° tilt and (**f**) cross-sectional SEM image of a single Si-MN.

Mask patterning is essential to make Si-MNs adequately reach target shapes. In the fabrication process, laser grooving was used to realize the orthogonal grid pattern opening of the SiO*_x_* protective layer on the wafer surface and to form deep kerfs in the wafer. Figure 2a shows the surface morphology of the wafer with the SiO*_x_* protective layer after laser grooving. The spacing between any two adjacent grooves is 500 µm. The corresponding enlarged image (Figure 2b) shows that the width of each groove is approximately 50 µm. This indicates that the mask pattern consists of small squares with an area of approximately 450 × 450 μm^2^. The depth of the kerfs directly affects the aspect ratio of the Si-MNs, which can be controlled by adjusting several parameter settings of the infrared fiber laser device, such as output power, scanning speed, duty ratio, and several laser scans. The depth of the laser kerfs in this study is approximately 90 µm, as shown in Figure 2c. After laser grooving, the Si-MN array was then fabricated through anisotropic etching in a 20 wt% KOH solution at 85 °C. In this procedure, the heights of the Si-MNs could be regulated by varying the depth of the laser kerfs and the duration of the alkaline etching. Figure 2d shows the morphology (30°-tilted SEM view) of the Si-MN array prepared after etching for 2 h. The spacing between two adjacent Si-MNs is also 500 μm, which corresponds to the spacing of the two adjacent laser grooves. A single Si-MN is shown in the enlarged SEM image, as shown in Figure 2e. The Si-MN exhibits good sharpness, which is conducive to penetrating the skin. The cross-sectional SEM image in Figure 2f shows that the Si-MN has an aspect ratio of approximately 2.4. The superior morphology of the Si-MNs serves as an excellent foundation for the subsequent production of BSi-MN arrays.

### 3.2. Influence of AgNO_3_/HF Ratio on BSi-MN Arrays

**Figure 3:**
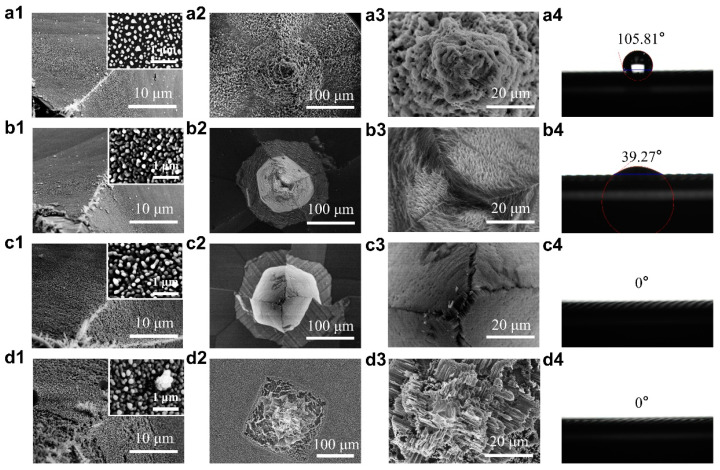
Top-view SEM images of Ag-NPs deposited on the surfaces of the Si-MNs in AgNO_3_/HF aqueous solution with Ag^+^ concentrations of (**a1**) 0.005 mol/L, (**b1**) 0.0075 mol/L, (**c1**) 0.01 mol/L, and (**d1**) 0.015 mol/L. (**a2**–**d2**) Top-view SEM images, (**a3**–**d3**) enlarged-view images, and (**a4**–**d4**) water contact angles of the surfaces of the corresponding BSi-MNs.

Subsequently, black silicon nanostructures were fabricated on the Si-MN arrays to yield BSi-MN arrays featuring hydrophilic surfaces through Ag-catalyzed chemical etching. To comprehensively understand the formation of the nanostructures on the Si-MN, we first investigated the influence of the deposition behavior of the Ag-NPs on the surface of the Si-MN patch on the formation of the nanostructures. The morphologies of the Ag-NPs deposited on the surface of the Si-MN patch in AgNO_3_/HF aqueous solution under different Ag^+^ concentrations and the corresponding black silicon nanostructures etched in HF/H_2_O_2_ aqueous solution are shown in Figure 3. To facilitate the study, we introduce the parameter

(1) $$\rho =\frac{c\left(HF\right)}{c\left(HF\right)+c\left(H_{2}O_{2}\right)}\times 100\%\;$$

where c(HF) and $c\left(H_{2}O_{2}\right)$ represent the molar concentrations of HF and H_2_O_2_ in the HF/H_2_O_2_ aqueous solution, respectively. At this stage, we fixed the HF concentration at 0.005 mol/L and the ρ value at 68%. When the Ag^+^ concentration was relatively low (0.005 mol/L), the size of the Ag-NPs was relatively dispersed (Figure 3(a1)), and typical porous black silicon structures appeared on the surface of the Si-MNs (Figure 3(a2,a3)). The corresponding contact angle of the surface of the BSi-MN array is 105.8° (Figure 3(a4)), indicating that it is still hydrophobic. When increasing the Ag^+^ concentration to 0.075 mol/L, the Ag-NPs grew and became dense (Figure 3(b1)), the black silicon nanostructures changed from porous structures to wispy nanowires (Figure 3(b2,b3)), and the corresponding surface contact angle decreased to 39.3° (Figure 3(b4)). A further increase in the Ag^+^ concentration to 0.01 mol/L resulted in larger and denser Ag-NPs (Figure 3(c1)) and more uniformly arranged nanowires (Figure 3(c2,c3)). The corresponding surface contact angle decreased to 0° (Figure 3(c4)), indicating an ultra-hydrophilic surface. When the Ag^+^ concentration reached 0.015 mol/L, the Ag-NPs continued to become larger and started to aggregate (Figure 3(d1)). However, the Si-MNs were severely etched away, destroying the original needle shape (Figure 3(d2,d3)). Although the surface contact angle was also 0°, the BSi-MNs were not suitable for TDD.

**Figure 4:**
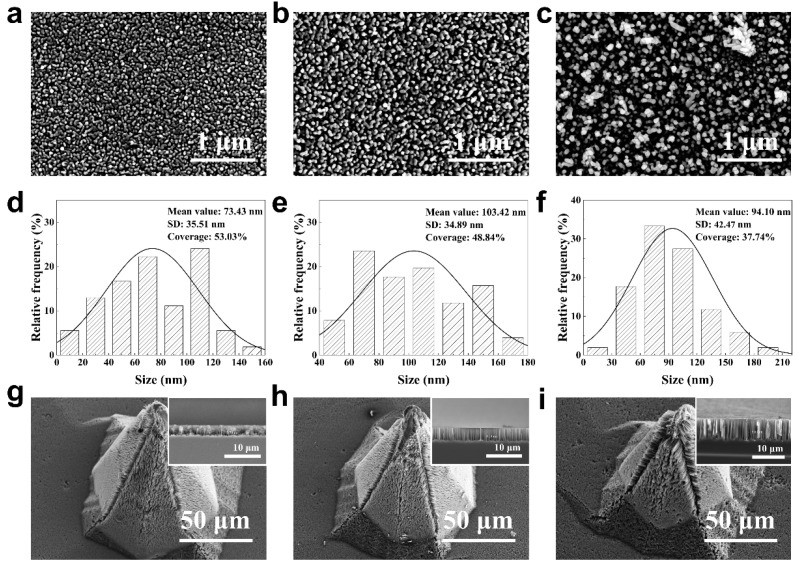
Top-view SEM images of Ag-NPs deposited on the surfaces of the Si-MNs in AgNO_3_/HF aqueous solution with HF concentrations of (**a**) 0.005 mol/L, (**b**) 0.009 mol/L, and (**c**) 0.014 mol/L. The corresponding statistical data of the Ag-NPs are shown in (**d**–**f**). (**g**–**i**) SEM images of the corresponding BSi-MNs at 30° tilt.

Previous studies have shown that the HF concentration in AgNO_3_/HF solutions is another important factor affecting the deposition behavior of Ag-NPs on silicon surfaces [45,46,47]. As well as removing the oxide layer, HF can form Si-F bonds on the silicon surface, which is conducive to the capture of holes and release of electrons [45], thus promoting the deposition of Ag-NPs. Figure 4a–c shows the morphologies of the Ag-NPs deposited on the surface of the Si-MNs produced by varying the HF concentration in the AgNO_3_/HF aqueous solution while maintaining the Ag^+^ concentration at the optimal 0.01 mol/L. The corresponding statistical analysis based on SEM images was obtained by using ImageJ (version 1.51j8) software, as shown in Figure 4d–f. The Ag-NPs deposited at an HF concentration of 0.005 mol/L were uniformly distributed, with a size distribution ranging from 10 to 150 nm. The corresponding mean value and standard deviation (SD) were 73 nm and 36 nm, respectively, indicating that the particle sizes were mainly distributed between 37 and 109 nm. With regard to the Ag-NPs deposited at an HF concentration of 0.009 mol/L, the particles started to agglomerate slightly, and the sizes were distributed from 50 to 170 nm. The corresponding mean value and SD were 103 nm and 35 nm, respectively, indicating that the particle sizes were mainly concentrated in the 68–138 nm range. When increasing the HF concentration to 0.014 mol/L, the particles exhibited significant agglomeration. In this case, the particle size distribution ranged from 15 to 195 nm with a mean value of 94 nm and SD of 43 nm. The coverage rate of Ag-NPs decreased with increasing HF concentration due to the agglomeration of particles.

Similarly, the Si-MN samples with the above three types of silver particles were etched in the HF/H_2_O_2_ aqueous solution with a ρ value of 68% to prepare the BSi-MN patches. The surface morphologies (30°-tilted SEM view) of the corresponding BSi-MNs are shown in Figure 4g–i. As can be seen, the tip and edge of the corresponding Si-MN were more severely etched in the HF/H_2_O_2_ aqueous solution with increasing HF concentration in the AgNO_3_/HF aqueous solution. This is because the agglomeration of Ag-NPs is more severe at the tip and edge (Figure S1, Supplementary Materials). The cross-sectional SEM images of the corresponding nanowire structures on the surfaces of the BSi-MNs are shown in the insets. The nanowire structures prepared by etching in the HF/H_2_O_2_ aqueous solution became longer and looser with increasing HF concentration in the AgNO_3_/HF aqueous solution. In the interim, it was observed that the water contact angles of the aforementioned BSi-MN patches were 0° (Figure S2, Supplementary Materials). Such an observation implies that the surface of BSi-MN evinces a remarkable degree of hydrophilicity.

### 3.3. Influence of H_2_O_2_/HF Ratio on BSi-MN Arrays

**Figure 5:**
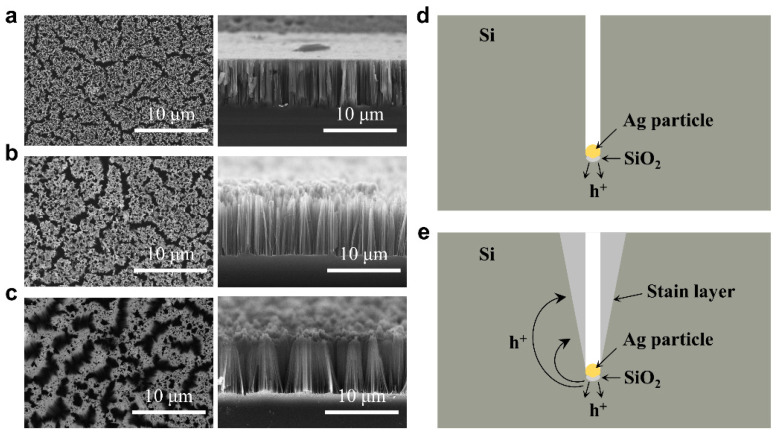
Top-view and cross-sectional SEM images of the nanowires prepared through Ag-catalyzed chemical etching on the surfaces of Si-MNs in HF/H_2_O_2_ aqueous solutions with different ρ values of (**a**) 88%, (**b**) 68%, and (**c**) 48%. Schematic diagrams of the mechanism of Ag-catalyzed chemical etching of silicon in (**d**) HF-rich and (**e**) H_2_O_2_-rich systems.

Through the above experiments and analyses, it is found that a suitable Ag^+^ concentration and low HF concentration are conducive to the uniform deposition of Ag-NPs, which is beneficial to better nanostructures for BSi-MNs. The next objective is to conduct a further investigation into the characteristics of Ag-catalyzed chemical etching on the surface of Si-MNs in the HF/H_2_O_2_ system. To do so, Ag-NPs were first deposited on the surfaces of the Si-MNs under the same conditions of a Ag^+^ concentration of 0.01 mol/L and HF concentration of 0.005 mol/L. The nanowires were then prepared through Ag-catalyzed chemical etching on the surfaces of Si-MNs in HF/H_2_O_2_ aqueous solutions with different ρ values. The top-view and cross-sectional SEM images of the as-prepared nanowires are shown in Figure 5. When the ρ value was 88%, nanopores rather than nanowires were observed on the surface, and the nanopores were perpendicular to the Si surface (Figure 5a). When decreasing the ρ value to 68%, distinct micropores appeared on the surface, corresponding to the “collapse” of some nanowires observed in the cross-sectional SEM image (Figure 5c). Further decreasing the ρ value to 48%, more and larger micropores appeared on the silicon surface, and the “collapse” phenomenon of nanowires was even more pronounced (Figure 5c).

Based on the aforementioned results, we proposed a mechanism for the Ag-catalyzed chemical etching of silicon in the HF/H_2_O_2_ system. It is well known that H_2_O_2_ functions as an oxidizing agent and preferentially injects holes into silicon through the Ag/Si interface. In a high ρ value (HF-rich) system, the concentration of H_2_O_2_ is relatively low, and the holes are only preferentially injected into the silicon at the bottom of the Ag-NPs. The silicon oxide at the bottom of the Ag particle is then etched by HF, and this process is repeated over and over again, which causes the Ag particle to move forward in the silicon (Figure 5d). In this process, the diameter of the etching path (pore) is comparable to the size of the Ag particle, which is due to the fact that the Ag-catalyzed chemical etching occurs only in a relatively small region in contact with the Ag particle. In a low ρ value (H_2_O_2_-rich) system, the concentration of H_2_O_2_ is relatively high, which can provide more hole injection. Apart from preferentially injecting silicon near the Ag particle through the Ag/Si interface [48], excess holes can diffuse into nearby silicon substrates and surfaces, causing these areas to be etched laterally to form stain layers [49]. The stain layers can dissolve in the HF/H_2_O_2_ mixture, resulting in the formation of inverted cone-shaped micropores (Figure 5e).

### 3.4. Properties of BSi-MN Arrays

**Figure 6:**
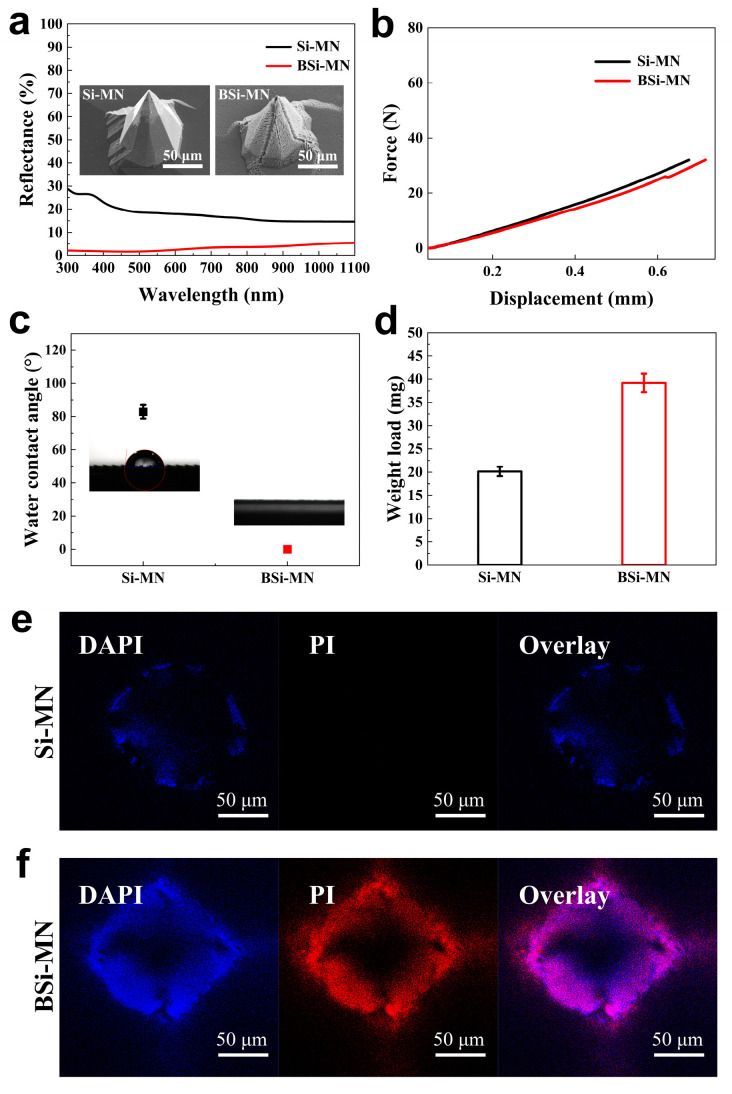
(**a**) Reflectance curve, (**b**) mechanical strength, (**c**) water contact angle, and (**d**) drug loading capacity of the BSi-MN samples, compared to those of the plain Si-MN samples. Test cases of antibacterial activity of (**e**) the Si-MNs and (**f**) BSi-MNs against Gram-negative *E. coli* using laser scanning confocal microscopy. Blue fluorescence indicates both live and dead *E. coli*, and red fluorescence indicates only dead *E. coli*.

Finally, the properties of the BSi-MNs prepared at a ρ value of 68% were evaluated and compared with typical Si-MNs. Photographs of the BSi-MN patch and Si-MN patch are shown in Figure S3 (Supplementary Materials). The BSi-MN patch shows a completely black appearance, indicating an extremely low surface reflection due to the strong light-trapping effect of the nanowire structures. As a comparison, the reflectance curves of the BSi-MN patch and Si-MN patch are shown in Figure 6a, and the corresponding SEM images are shown in the inset. To determine whether the black silicon nanowire structures could potentially compromise the mechanical strength of the MNs, compression experiments were conducted on both plain Si-MNs and BSi-MNs. It was found that the mechanical properties of the BSi-MNs exhibited similar mechanical strength to that of the plain Si-MNs (Figure 6b), and no fracture was observed during the test. The results indicate that the black silicon nanowire structures have no significant effect on the mechanical strength of Si-MNs.

Figure 6c presents the water contact angles of the plain Si-MN patch and BSi-MN patch. The water contact angle of the Si-MN patch is approximately 82.82°, while that of the BSi-MN patch is 0°. This indicates that the BSi-MN patch has an ultra-hydrophilic surface, which is in line with our expectations. To determine the actual drug loading capacity of the BSi-MN patch, weighing experiments were conducted on the Si-MN patches and BSi-MN patches with an area of 2 × 2 cm^2^. Specifically, normal saline was first added to the surfaces of both patches using a syringe until excess, and the patches were then gently vertically lifted to remove excess normal saline. The weight of the MN sample is subtracted from the weight obtained by weighing to obtain its actual loading capacity. The weights of the two types of MN patches loaded with normal saline are shown in Figure 6d. The drug loading of the BSi-MN patch is twice that of the plain Si-MN patch with the same area. To ensure the accuracy of weighing, each sample was weighed at least five times, and the average value of the final result was taken.

### 3.5. Preliminary Antibacterial Experiments on BSi-MN Arrays with E.coli

In addition, we evaluated the antibacterial activity of the BSi-MNs against Gram-negative *E. coli* using plain Si-MNs as the control group. To quantitatively compare the antimicrobial performance of the two types of MNs, *E. coli* was cultured separately on the two patches. After staining with live/dead cells, fluorescence images were obtained under laser scanning confocal microscopy, as shown in Figure 6e,f. The first channel is the blue channel, which corresponds to all bacteria (both live and dead) on the sample. The second channel is the red channel, which corresponds to dead bacteria. For the BSi-MN sample, more red fluorescence was observed compared to the reference Si-MN sample, indicating that more bacteria were killed. This suggests that the nanowire structures on the surface of the BSi-MNs can enhance the antibacterial activity, which may be due to the stretching effect of the nanowire structure on bacterial cell membranes. The stretching of the cell wall adsorbed on the nanowire structures is due to the interaction between the elastic deformation of the cell wall and the intrinsic attraction of the cell wall to the surface. The mechanical bactericidal effect on the surfaces of the BSi-MNs is associated with the stretching of the cell membrane beyond their elastic limit. The cell membrane ruptures in the suspension region between the nanowires when the bacterial cells are adsorbed onto the nanowires [50,51]. The high-aspect ratio nanostructures possess a unique capability of storing elastic energy through their flexibility. When these nanostructures come into contact with bacterial cells, the elastic energy that was previously accumulated in the nanowires is discharged, causing the nanowires to bend and physically perturb the cell membrane by stretching it, ultimately resulting in cell death [52,53]. It is conceivable that the same mechanism of action would occur when applied to the skin to prevent bacteria from growing in the pierced skin area. The results indicate that the BSi-MNs also have potential in antibacterial applications.

## 4. Conclusions

In summary, we propose a method to prepare a novel BSi-MN array with ultra-hydrophilic surfaces for high drug loading by using simple laser patterning, standard alkaline etching, and well-established Ag-catalyzed chemical etching. The preparation parameters of black silicon nanostructures on plain Si-MNs were investigated in detail. The experimental results show that a suitable Ag^+^ concentration, relatively low HF concentration, and moderate ρ value are suitable for the preparation of ideal BSi-MNs. The mechanical strength of the as-prepared BSi-MNs is close to that of plain Si-MNs, which is beneficial for practical skin piercing applications. Moreover, the BSi-MNs exhibit excellent drug-loading capability and certain antimicrobial activity. Taken together, our fabrication method for BSi-MNs is simple and can facilitate manufacturing in a large area and on a large scale, which has great application potential in the TDD field.

## Data Availability

The original contributions presented in the study are included in the article and the Supplementary Materials. Further inquiries can be directed to the corresponding authors.

## Supplementary Materials

The following supporting information can be downloaded at: https://www.mdpi.com/article/10.3390/jfb14050245/s1, Figure S1: SEM images of Ag-NPs deposited on the surfaces of Si-MNs in AgNO_3_/HF aqueous solutions with HF concentrations of (a) 0.005 mol/L, (b) 0.009 mol/L, and (c) 0.014 mol/L; Figure S2: Water contact angles of the surfaces of the corresponding BSi-MNs at HF concentrations of (a) 0.005 mol/L, (b) 0.009 mol/L, and (c) 0.014 mol/L; Figure S3: Photographs of the Si-MN patch (left) and BSi-MN patch (right).
