# Supplementary material for: Fabrication of Black Silicon Microneedle Arrays for High Drug Loading
Source: J Funct Biomater. 2023 Apr 26;14(5):245. doi: 10.3390/jfb14050245 (PMC10219322; doi:10.3390/jfb14050245)
Supplement: Supplementary file 1 [file jfb-14-00245-s001.zip › jfb-2346103-supplementary.docx]

**Supporting information**

**Fabrication of black-silicon microneedle array****s** **for high drug loading**

Wei Cheng^1.†^, Xue Wang^2,†^, Shuai Zou^1,3,*^, Mengfei Ni^1^, Zheng Lu^1^, Longfei Dai^1^, Jiandong Su^2^, Kai Yang^1,*^, Xiaodong Su^1^

^1^ Jiangsu Key Laboratory of Thin Films, School of Physical Science and Technology, Soochow University, Suzhou 215006, China; 20204208004@stu.suda.edu.cn (W.C.); mfni@stu.suda.edu.cn (M.N.); luzh@suda.edu.cn (Z.L.); 20227908001@stu.suda.edu.cn (L.D.); xdsu@suda.edu.cn (X.S.)

^2^ Department of Burn and Plastic Surgery, Suzhou Hospital Affiliated to Nanjing Medical University, Suzhou 215000, China; 2022121959@stu.njmu.edu.cn (X.W.); jiandongsu@njmu.edu.cn (J.S.)

^3^ Suzhou Xiangbang Biotechnology Co., LTD. Suzhou 215006, China

* Correspondence: [szou@suda.edu.cn](mailto:szou@suda.edu.cn) (S.Z.); yangkai@suda.edu.cn (K.Y.)

† These authors contributed equally to this work.


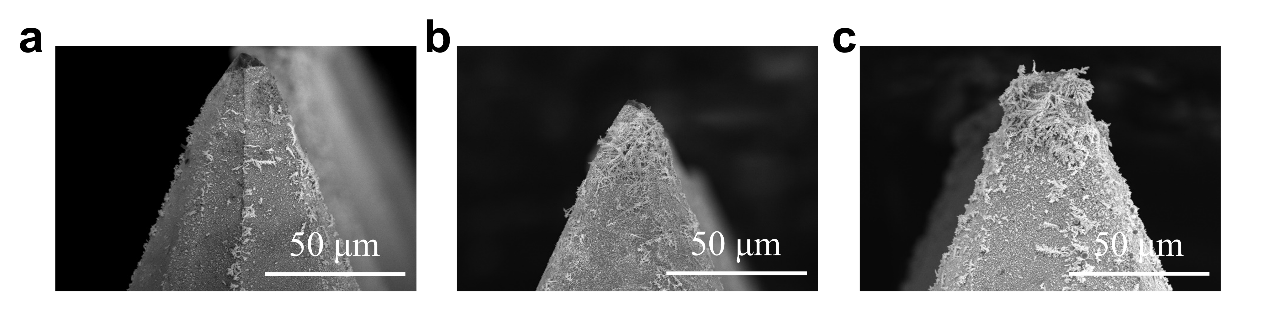


**Figure S1.** SEM images of Ag-NPs deposited on the surfaces of Si-MNs in AgNO_3_/HF aqueous solutions with HF concentrations of (a) 0.005 mol/L, (b) 0.009 mol/L, and (c) 0.014 mol/L.


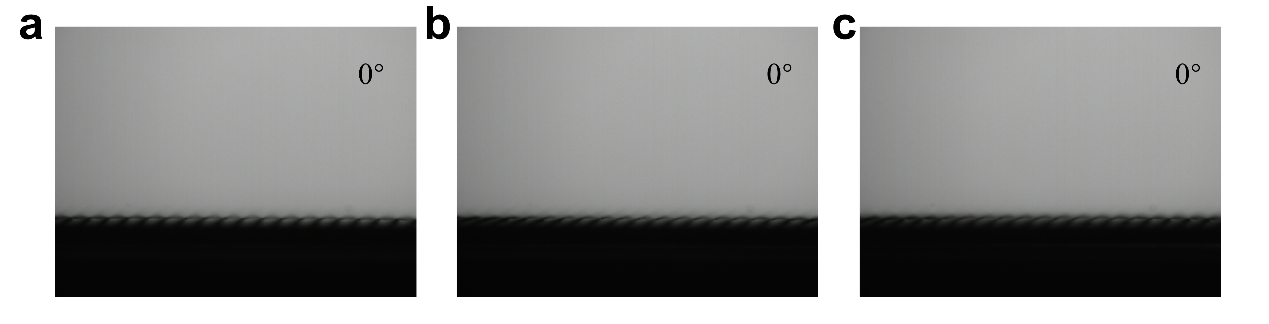


**Figure S2.** Water contact angles of the surfaces of the corresponding BSi-MNs at HF concentrations of (a) 0.005 mol/L, (b) 0.009 mol/L, and (c) 0.014 mol/L.


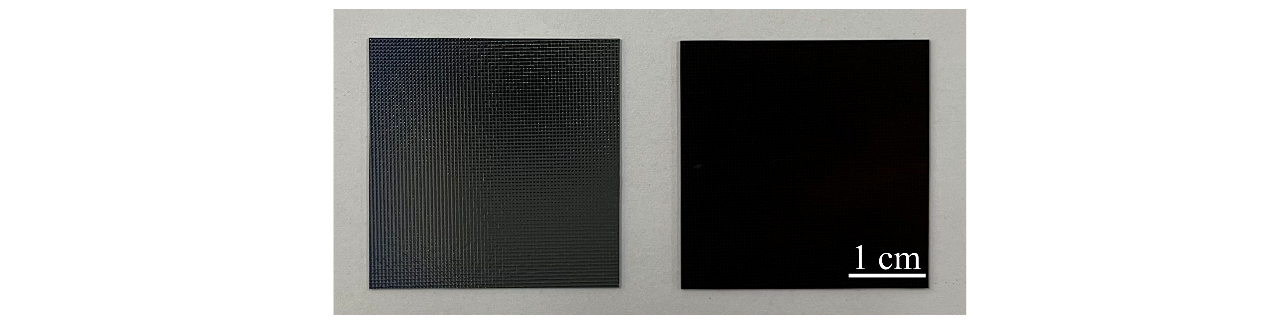


**Figure S3.** Photographs of the Si-MN patch (left) and BSi-MN patch (right).
